# Life-Threatening Bleeding from Peristomal Varices after Cystoprostatectomy: Multimodal Approach in a Cirrhotic, Encephalopathic Patient with Severe Portal Hypertension

**DOI:** 10.1155/2015/785010

**Published:** 2015-01-29

**Authors:** Sergej E. L. Staubli, Tobias Gramann, Christoph Schwab, David Semela, Lukas Hechelhammer, Daniel S. Engeler, Hans-Peter Schmid, Dominik Abt, Livio Mordasini

**Affiliations:** ^1^Department of Urology, Cantonal Hospital St. Gallen, 9007 St. Gallen, Switzerland; ^2^Department of Gastroenterology and Hepatology, Cantonal Hospital St. Gallen, 9007 St. Gallen, Switzerland; ^3^Department of Radiology and Nuclear Medicine, Cantonal Hospital St. Gallen, 9007 St. Gallen, Switzerland

## Abstract

The bleeding of peristomal varices due to a portosystemic shunt is rare but potentially life-threatening in cirrhotic patients with portal hypertension. The scarce case reports in the literature recommend transjugular intrahepatic portosystemic shunt (TIPS) to prevent further bleeding. We report on a 72-year-old man who was referred to our hospital because of life-threatening bleeding from peristomal varices, three years after radical cystoprostatectomy for invasive bladder cancer. CT imaging showed liver cirrhosis with a prominent portosystemic shunt leading to massively enlarged peristomal varices. TIPS was taken into consideration, but not possible due to hepatic encephalopathy (HE). Medical therapy with lactulose and the nonselective beta-blocker carvedilol was initiated to treat HE and portal hypertension. In a second step, the portosystemic shunt was percutaneously embolized. Here, we present a multimodal approach to treat intractable bleeding from peristomal varices in a patient with ileal conduit urinary diversion, not suitable for TIPS.

## 1. Introduction

Peristomal varices represent ectopic portosystemic shunts due to portal hypertension, which is the major complication in liver cirrhosis. Bleeding from peristomal varices is rare and challenging to diagnose, but potentially life-threatening in cirrhotic patients, and deaths from exsanguination have been reported. The majority of collateral circulation in portal hypertension arises from esophageal or gastric varices. Ectopic bypasses such as stomal varices cause only 1–5% of all variceal bleeding episodes [1]. The site of bleeding is usually located at the vulnerable mucocutaneous junction of the stoma, between the high-pressure portal system and the low-pressure systemic venous system [2]. Since the first report of peristomal haemorrhage in 1968, different therapies such as sclerotherapy, embolization, transjugular intrahepatic portocaval shunt (TIPS), or liver transplantation have been used to treat and prevent variceal bleeding.

The scarce case reports in the literature of patients with ureteroileal conduit and ectopic variceal bleeding unanimously recommend TIPS to prevent further bleeding [3, 4]. TIPS is an artificial portovenous shunt within the liver that lowers the pressure in the portal vessels. However, portocaval shunting reduces the hepatic clearance of ammonia leading to hepatic encephalopathy (HE) with neurocognitive impairment, stupor, and coma in extreme cases. Insertion of a TIPS can aggravate the preexisting HE in cirrhotic patients and was therefore contraindicated in our patient. Here, we present, to the best of our knowledge, the first report of a multimodal approach to treat intractable bleeding from peristomal varices in a patient not suitable for TIPS.

## 2. Case Report

A 72-year-old man was referred to our hospital because of life-threatening bleeding from peristomal varices, 3 years after radical cystoprostatectomy for muscle invasive bladder cancer. In the past few months, the patient had been assessed in our outpatient clinic for multiple episodes of “macrohaematuria.” Ileal looposcopy with retrograde nephroureterography was without pathological findings.

At admission, the patient showed massive hemodynamically relevant peristomal haemorrhage with haemoglobin of 27 g/L. The patient could be successfully resuscitated after transfusion of seven units of red blood cells reaching a haemoglobin value of 82 g/L.

**Figure 1:**
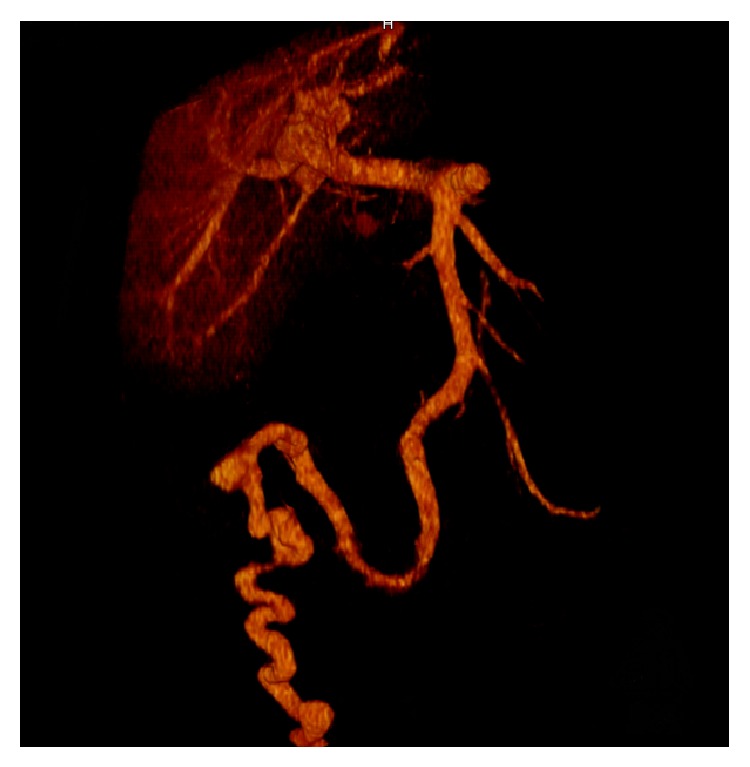
Computed tomography 3D reconstruction illustrating the portosystemic shunt.

**Figure 2:**
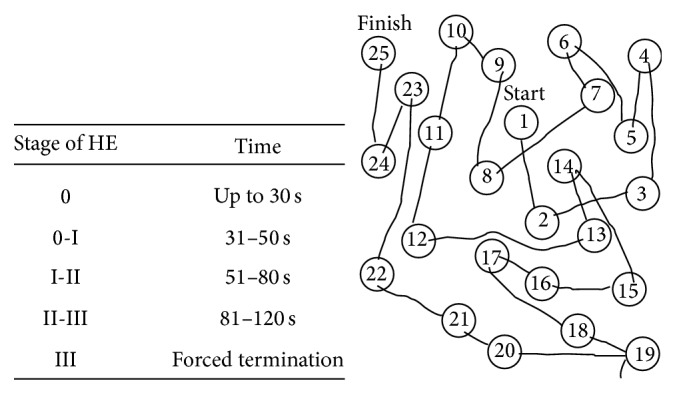
Connect-the-numbers test to assess the grade of hepatic encephalopathy.

**Figure 3:**
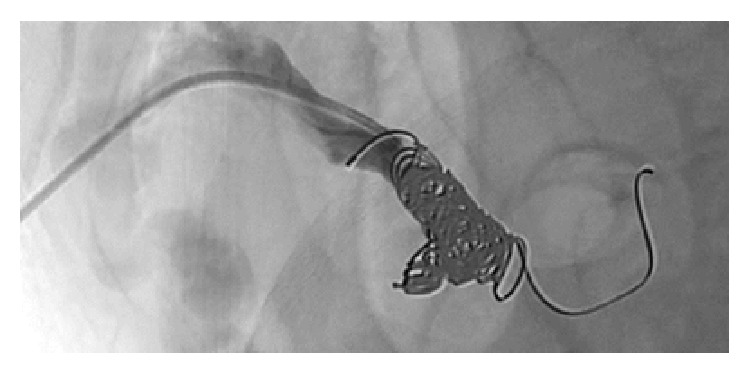
Angiography after coiling of the portosystemic shunt.

CT imaging showed features of liver cirrhosis with a prominent portosystemic shunt (Figure 1), leading to massively enlarged peristomal varices and excluded upper urinary tract bleeding. TIPS was taken into consideration as a secondary prophylaxis to prevent rebleeding. However, neurologic assessment with connect-the-numbers test (Figure 2) revealed clinically relevant HE, making the patient not suitable for TIPS. Medical therapy with lactulose and the nonselective beta-blocker carvedilol was initiated to treat HE and portal venous hypertension, respectively. In a second step, the portosystemic shunt was percutaneously embolized with a combination of coils and histoacryl/lipiodol (Figure 3). Intravenous manometric measurement confirmed substantial portal hypertension with a portoatrial pressure gradient of 15 mmHg (normal 3–9 mmHg) before embolization.

The postoperative course was uneventful and the patient could be discharged from hospital without further bleeding. Ultrasound revealed* de novo* ascites in the first follow-up control 1 week after the embolization, most likely due to a slight aggravation of portal hypertension after percutaneous embolization of the shunt. Treatment with spironolactone (100 mg/d) was initiated. The final follow-up visit was done 8 weeks after the embolization and showed complete remission of ascites. The patient was asymptomatic and without rebleeding.

## 3. Discussion

Most patients with peristomal varices have multiple hemorrhagic episodes that require repeated hospital admissions. The main goal after resuscitation and successful treatment of active variceal bleeding is the prevention of further bleeding by reduction of portal hypertension and/or embolization of the varix. Portal hypertension is defined as elevation of hepatic venous pressure gradient to >5 mmHg. In gradients greater than 10 mmHg, blood flow through the hepatic portal system is redirected with the consecutive development of portosystemic shunts. The most effective measure to reduce portal hypertension is TIPS, an intervention first described in 1969 by Rösch et al. TIPS decreases vascular resistance of the liver, leading to a reduction of portal venous pressure, and bleeding from friable variceal tissue is less likely to occur [5]. However, portocaval shunting reduces the first-pass clearance of ammonia, increasing the risk for HE. Thus, exclusion of HE is mandatory before assigning a patient to TIPS. The severity of HE is graded to 4 stages according to the West Haven Criteria (Table 1), based on the impairment of autonomy, changes in consciousness, intellectual function, behavior, and the dependence on therapy [6]. The connect-the-numbers test represents a validated and sensitive psychodiagnostic test for quantitative detection of HE [7]. Numbers arranged in an arbitrary sequence have to be connected with one another as quickly as possible in their correct sequence by using a pencil to draw a line between them. A healthy individual will always be able to perform this task in less than 30 seconds. Our patient, however, required 132 seconds, indicating severe HE.

Treatment of HE relies on suppressing the production of ammonia in the intestine, commonly done with the laxative lactulose. The disaccharide is not absorbed from the digestive tract and decreases the generation of ammonia by bacteria, by converting ammonia to nonabsorbable ammonium (NH_4_). Doses of 15–30 mL are administered three times a day. In addition, selective antibiotic gut decontamination with rifaximin (550 mg/d) can be used to reduce ammonia production.

Variceal bleeding can be prevented with a nonselective beta-blocker such as carvedilol. It has been shown that reduction of hepatic venous pressure gradient of at least 20% was associated with a marked reduction of the risk of variceal bleeding [8]. Recently, interventional radiologic procedures such as the percutaneous embolization were introduced to treat ectopic variceal bleeding locally via direct percutaneous coil embolization of the stomal varix in patients with a high risk of HE after TIPS [9]. This procedure is technically less demanding and less invasive than a TIPS procedure. On the other hand, portal hypertension is not improved and often even aggravated leading to a higher rebleeding risk than after TIPS [10].

In conclusion, ectopic variceal bleeding has to be taken into consideration in patients with ileal conduit, hepatic dysfunction, and recurrent episodes of “macrohaematuria.” TIPS represents the most effective method to reduce portal hypertension and to prevent rebleeding. HE has to be excluded before referring patients to TIPS. The connect-the-numbers test represents an easy to perform validated diagnostic tool to exclude or detect HE. Multimodal treatment with lactulose, rifaximin, carvedilol, and percutaneous embolization represent possible therapeutic options in patients with intractable, recurrent bleeding from peristomal varices not suitable for TIPS. After percutaneous embolization portal hypertension can be aggravated and* de novo* development of ascites has to be excluded at follow-up visits.

## Figures and Tables

**Table 1:** Hepatic encephalopathy scoring algorithm.

Stage of HE	Abnormalities
1	Mild changes in personality and thinking
2	Sleepiness, increased confusion
3	Asleep most time
4	Coma

## Abbreviations

TIPS: Transjugular intrahepatic portosystemic shunt
HE: Hepatic encephalopathy
CT: Computed tomography.
